# Epidemiological Study and Seropositivity in Viral Panel Testing Among Corneal Tissue Donors

**DOI:** 10.7759/cureus.69728

**Published:** 2024-09-19

**Authors:** Nemi Isabel Pérez Peña, Luis Enrique Sanchez García, José Manuel García Romero, Alberto Montoya Vázquez, Karen Alonso Aguiñiga, Floricel O Villegas Amador, Daniela Esperanza Tolentino Pérez, Lilia Yolanda Ochoa Cisneros, Jesus A Diaz Ugalde, Irene García Hernández

**Affiliations:** 1 Transplant and Donation Department, Regional General Hospital 1 of the Mexican Social Security Institute, Querétaro, MEX; 2 General Practice, Autonomous University of Querétaro, Querétaro, MEX

**Keywords:** comorbidities, cornea, corneal procurement, donors, epidemiology, tissue donor, viral panel

## Abstract

Background: Corneal tissue is a critical component for vision restoration through transplantation, yet the availability of suitable corneal tissue is limited. This limitation results in long waiting lists and high demand, especially in countries with lower donation rates compared to global benchmarks. In Mexico, the corneal donation rate remains significantly behind other Latin American countries and leading nations such as Spain. Understanding the characteristics of corneal tissue donors is essential for improving donation practices and addressing the shortage of available tissues.

Materials and methods: This descriptive retrospective study analyzed electronic medical records of cadaveric corneal donors at the General Regional Hospital 1 of the Mexican Social Security Institute of Querétaro from January 2022 to December 2023. Donors were included based on criteria such as age (2-85 years) and known cause of death. Exclusion criteria included age outside the specified range, neoplastic diseases, active systemic infections, and prior ocular surgeries. Serological testing for human immunodeficiency virus (HIV), Venereal Disease Research Laboratory (VDRL), coronavirus disease 2019 (COVID-19), hepatitis B virus (HBV), and hepatitis C virus (HCV was performed to assess viral reactivity. Statistical analyses were conducted using IBM SPSS Statistics for Windows, Version 25 (Released 2017; IBM Corp., Armonk, New York, United States), summarizing data with descriptive statistics.

Results: The study included 185 corneal donors with a mean age of 56.34 years. The majority were male (109; 58.9%), and the leading causes of death were cardiogenic shock (34; 18.4%), hypovolemic shock (31; 16.8%), and acute respiratory failure (30; 16.2%). Exclusion due to positive serological tests included seven donors (3.8%) for HIV and seven (3.8%) for SARS-CoV-2. A total of 16 donors (8.6%) were excluded due to positive results in the viral serological panel. The most common comorbidities were chronic kidney disease (36; 19.5%), type 2 diabetes mellitus (28; 15.1%), and systemic hypertension (31; 16.8%).

Conclusions: The study highlights a predominantly male donor profile with a mean age of 56.34 years and emphasizes cardiogenic shock as a leading cause of death. The notably higher seropositivity rate of 8.6% for various viral infections compared to international reports indicates a need for improved health interventions and screening processes. The focus on cardiovascular and respiratory causes of death underscores regional health issues affecting donation patterns. To address the organ and tissue donation shortfall, it is crucial to enhance coordination within donation teams and increase public awareness, given the significant gap in donation rates compared to leading countries.

## Introduction

The cornea ranks second among patients on transplant waiting lists, only behind kidneys, resulting in high national demand for this tissue, as corneal transplants significantly improve recipients' quality of life. However, donation rates in our country are low compared to other Latin American countries and the rest of the world. The national donation rate between 2007 and 2017 was 3.2 donations per million people (pmp) in 2007, increasing to 3.94 pmp in 2017. Unfortunately, these figures are low compared to donation statistics from other Latin American countries such as Argentina (13.4 pmp), Chile (9.6 pmp), Brazil (16.3 pmp), Colombia (8.9 pmp), Cuba (12.3 pmp), and Uruguay (18.9 pmp), and are far from leading countries like Spain, which has a rate of 47.0 pmp [1,2].

As of 2024, there is a long waiting list for corneal transplants, currently comprising 3,338 patients, which represents a 21.7% decrease from 4,267 in 2022 [3]. During 2022 and 2023, a total of 2,007 and 2,681 donations from deceased individuals were completed, respectively. Querétaro ranks third in completed donations from deceased individuals nationally, behind the State of Mexico and Mexico City, and holds the seventh and eighth positions for completed donations due to brain death during 2022 and 2023 [4].

To ensure proper transplantation, the quality of the donated tissue is paramount; therefore, donors must meet strict inclusion and exclusion criteria. Age is one such criterion, with a minimum of two years and a maximum of 85 years established [5]. The time between death and tissue retrieval significantly impacts corneal tissue viability, which should not exceed six hours, although this period can be extended to 12 hours if the body is in optimal refrigeration conditions [6]. Some conditions that prevent donation include test positive for a viral panel of blood-borne or sexually transmitted diseases, septicemia, a history of previous intraocular surgery, intrinsic ocular diseases (such as retinoblastoma, keratoconus, keratoglobus, etc.), histoplasmosis, and cytomegalovirus [7,8].

The impact of corneal transplants is significant, allowing for the recovery of visual function and immediate improvement in quality of life. It is a low-cost and quick procedure, averaging 40 minutes, and does not require systemic immunosuppressive therapy, with a 95% success rate [9]. As of today, there are a total of 3,338 patients on the corneal tissue waiting list. This list has been decreasing over the years since 2015-2016, when the National Transplant Center (CENATRA) records showed a total of 7,486 patients waiting [3].

The cornea is an essential component for vision. Corneal transplants or keratoplasties are vital procedures for restoring vision in patients with various corneal conditions. However, the availability of suitable corneal tissue for transplantation is limited, resulting in a long waiting list of patients [10]. Furthermore, there is a lack of comprehensive studies analyzing the clinical and epidemiological characteristics of corneal tissue donors in Mexico and other Latin American countries, unlike other regions of the world where the epidemiological and clinical characteristics of donor populations are well understood. Understanding these characteristics is crucial for determining the suitability of donated tissue for transplantation and identifying potential factors that may affect tissue quality and, consequently, the success of corneal transplants.

## Materials and methods

**Figure 1:**
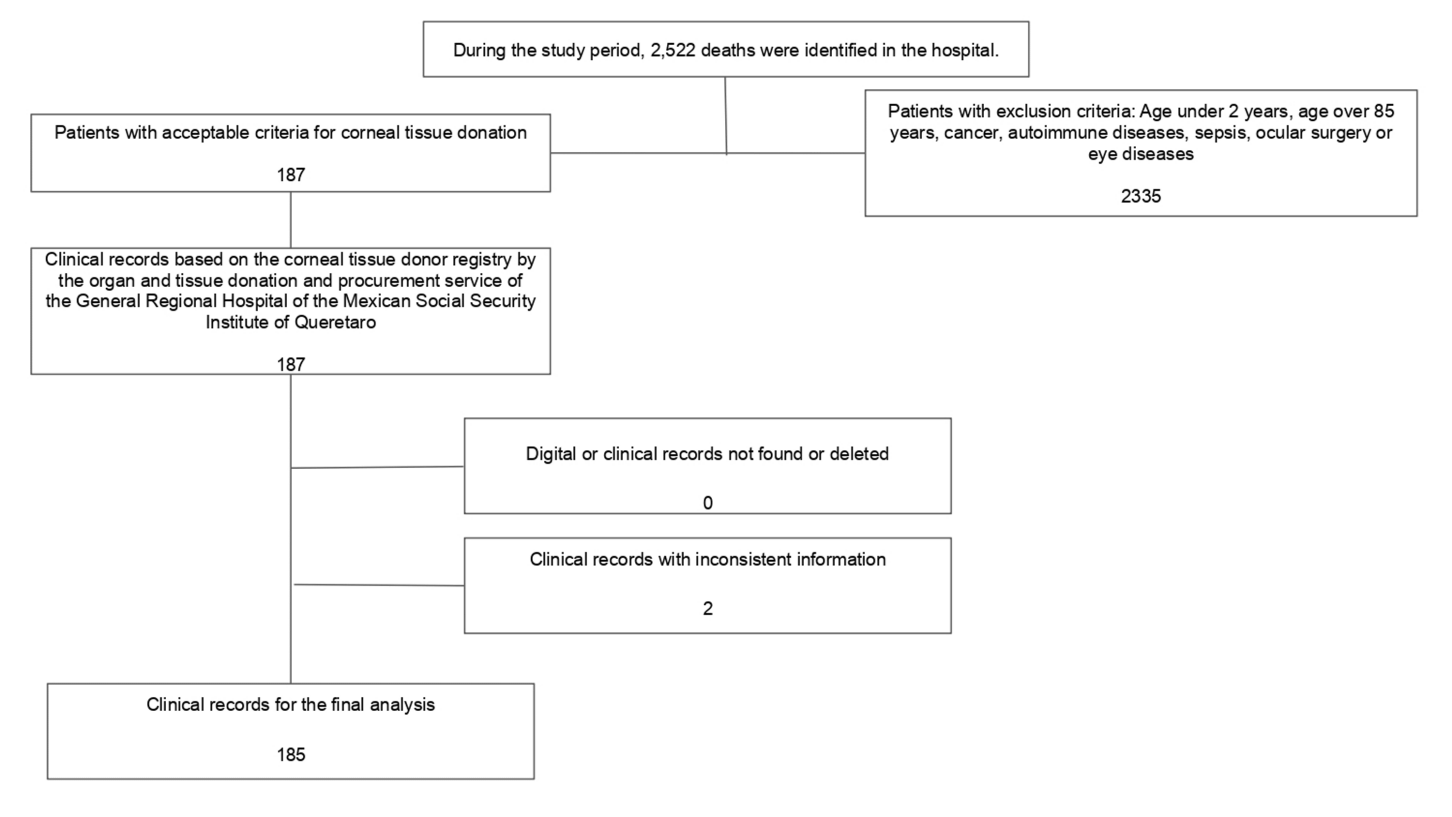
Flowchart of selection criteria for final study sample The number of patients selected as well as those excluded from the study based on the eligibility criteria for corneal tissue donation are shown. Additionally, incomplete and inconsistent medical records were excluded

This descriptive retrospective study analyzed electronic medical records of patients who participated as cadaveric corneal donors at General Regional Hospital 1 of the Mexican Social Security Institute of Querétaro from January 2022 to December 2023. The inclusion criteria for corneal tissue donors were as follows: age greater than 2 years and less than 85 years, a known cause of death, and time of death less than two hours. Exclusion criteria included age less than 2 years or greater than 85 years, neoplastic disease, active systemic infection, history of autoimmune disease, corneal injury, and previous ocular surgery (Figure 1). 

The study utilized a convenience sampling method. We included all corneal tissue donations that met the eligibility criteria and had obtained informed consent from the donors' families. This approach was based on the availability of cases that met predefined requirements during the study period, rather than employing a random or probabilistic sampling technique.

After corneal tissue extraction, all patients, with prior consent from their families, underwent serological testing for viral panel reactivity, including human immunodeficiency virus (HIV), Venereal Disease Research Laboratory (VDRL), coronavirus disease 2019 (COVID-19), hepatitis B virus (HBV), and hepatitis C virus (HCV). This testing was part of the corneal procurement protocol to ensure that there was no risk of infectious contagion for the corneal tissue recipients.

Donor characteristics were categorized nonexclusively, allowing for the documentation of multiple characteristics per patient where applicable. This approach ensured that all relevant data regarding donor characteristics were comprehensively captured.

The donation request was made to the patient's family, and approval for participation in the protocol was also sought. Subsequently, the Local Health Research Committee 2201 and the Research Ethics Committee No. 22018 approved the study protocol.

Statistical analysis

Data were collected over a six-month period and included the following variables: age, sex, comorbidities, cause of death, cardiac arrest, brain death, HIV seropositivity, positive VDRL, hepatitis C seropositivity, and hepatitis B seropositivity.

Statistical analyses were conducted using IBM SPSS Statistics for Windows, Version 25 (Released 2017; IBM Corp., Armonk, New York, United States). A descriptive statistical approach was used to summarize the data, which included calculating percentages for categorical variables and means ± standard deviations (SD) or medians (IQR) for continuous variables, as appropriate for the data distribution.

## Results

In the studied period, a total of 185 corneal donors were registered at General Regional Hospital 1 in Querétaro. Among these, 14 (7.57%) were multiorgan donors and 171 (92.4%) were cardiac arrest donors. 

**Figure 2:**
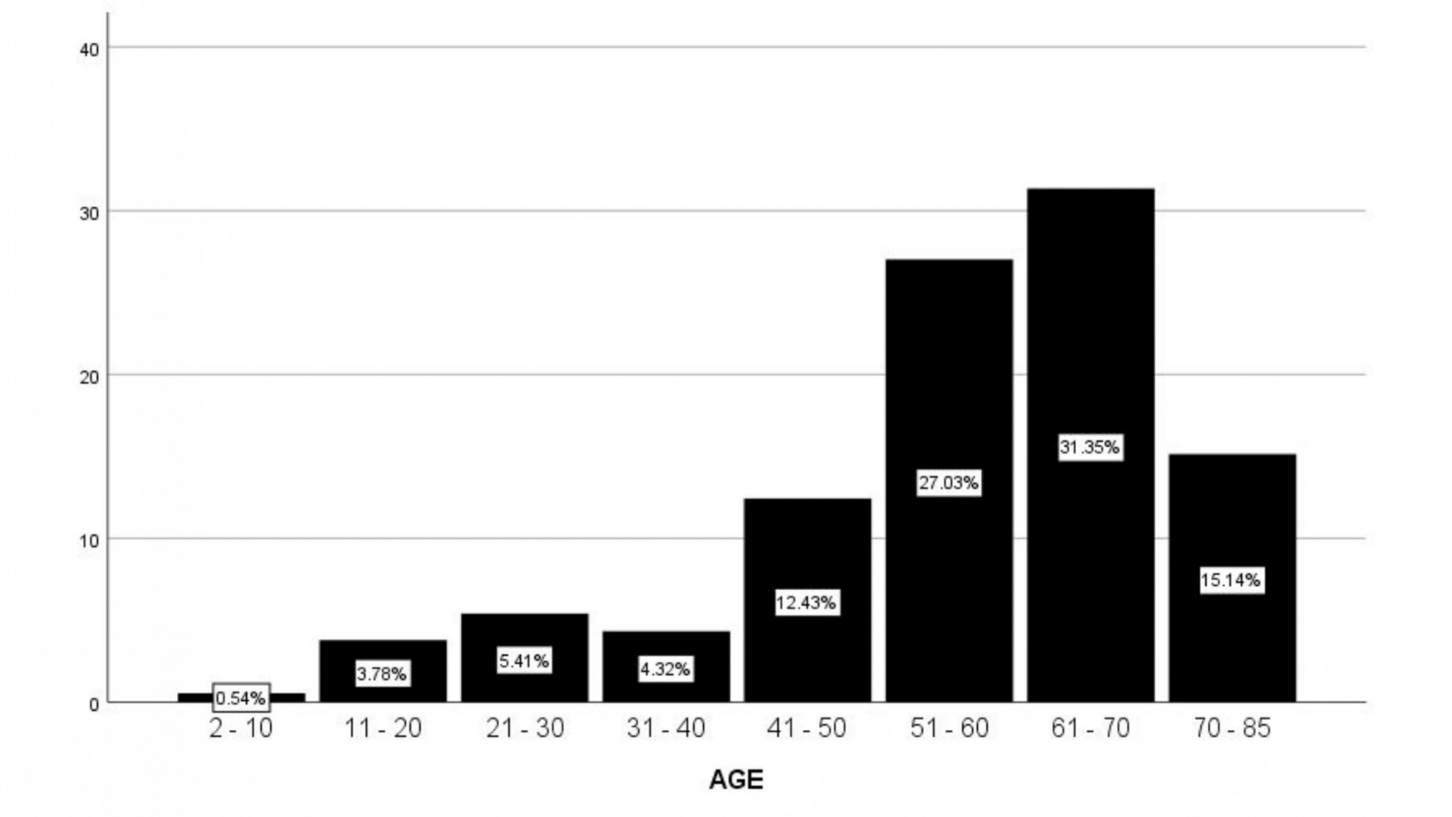
Age ranges of corneal donors The horizontal axis shows the different age groups, and the vertical axis represents the percentage of the total that these groups account for

The age range of donors spanned from two to 85 years, with a mean age of 56.34 years. The distribution by age groups was as follows: 2-10 years (1; 0.54%), 11-20 years (7; 3.78%), 21-30 years (10; 5.41%), 31-40 years (8; 4.32%), 41-50 years (23; 12.43%), 51-60 years (50; 27.03%), 61-70 years (58; 31.35%), and 71-85 years (28; 15.14%) (Figure 2).

**Figure 3:**
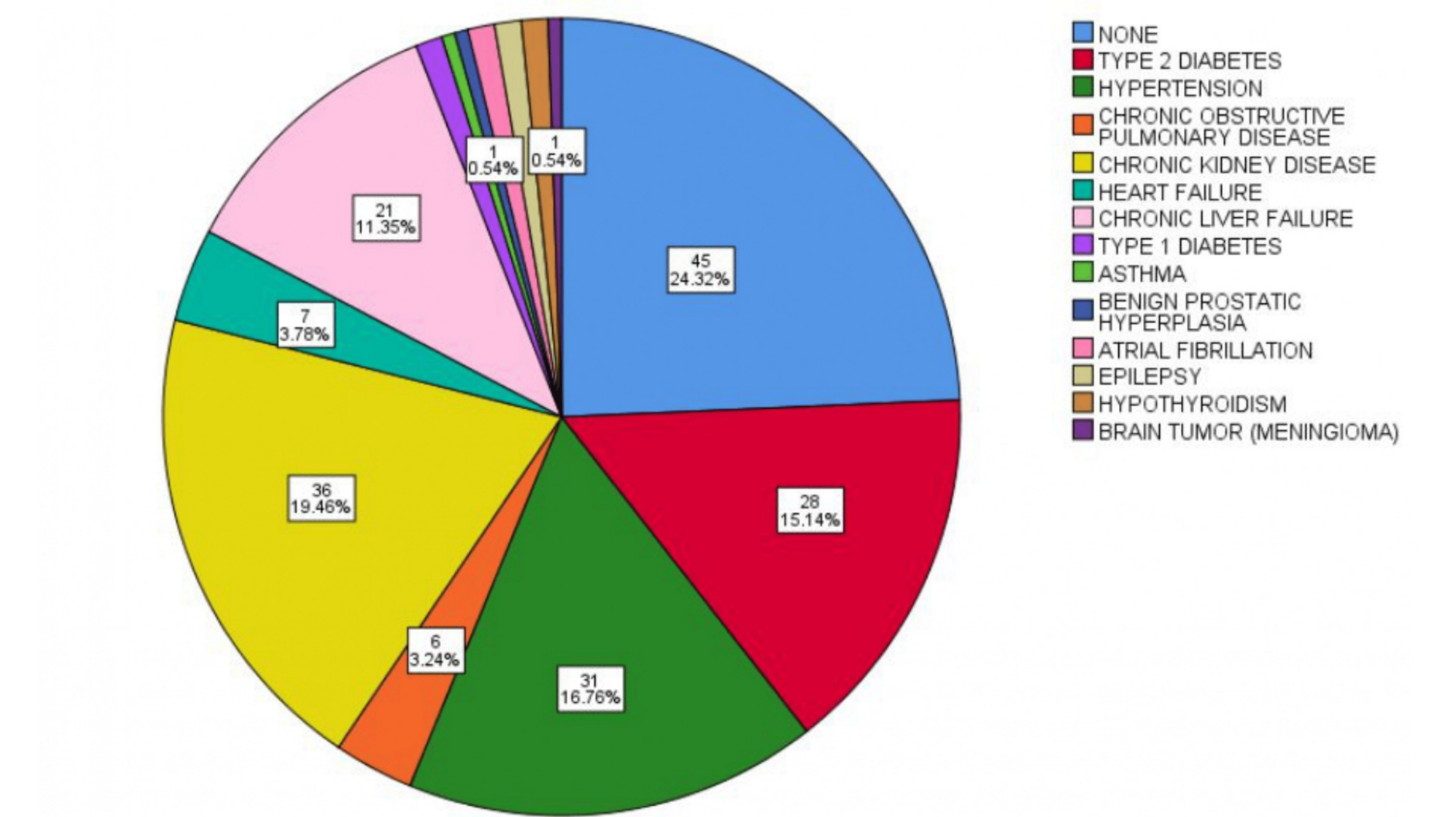
Main comorbidities among corneal tissue donors A pie chart is shown displaying both the frequency (n) and the percentage (%) of each comorbidity present among the corneal donors

Of the 185 corneal donors, 109 were men (58.9%) and 76 were women (41.1%). Among the corneal donors, 45 (24.3%) had no comorbidities. The most common comorbidities were chronic kidney disease (36; 19.5%), type 2 diabetes mellitus (28; 15.1%), systemic hypertension (31; 16.8%), chronic liver failure (21; 11.5%), heart failure (7; 3.8%), chronic obstructive pulmonary disease (6; 3.2%); hypothyroidism, type 1 diabetes, and atrial fibrillation (2; 1.1% each); and asthma, benign prostatic hyperplasia, and epilepsy (1; 0.5% each) (Figure 3).

**Figure 4:**
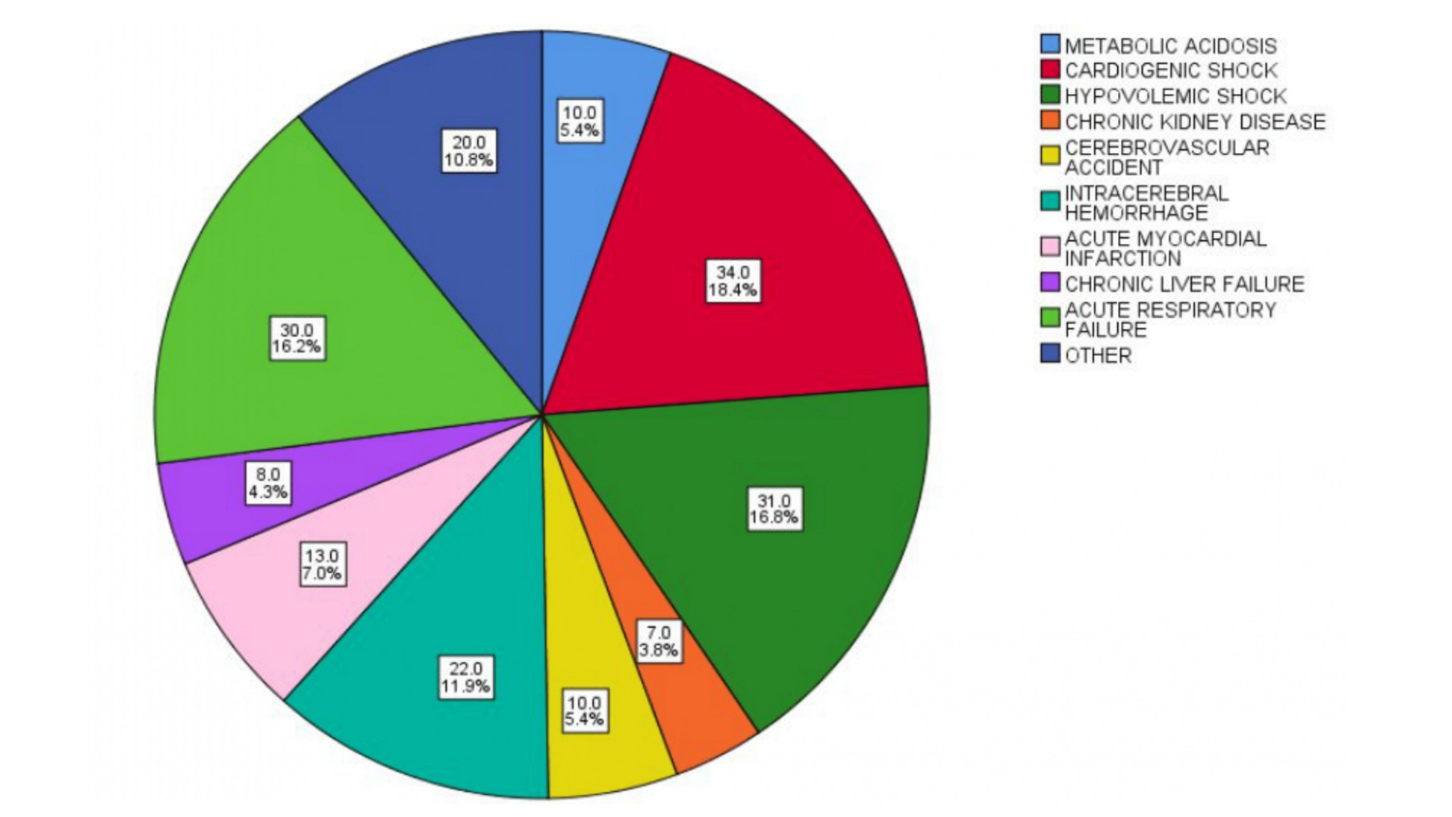
Main causes of death among corneal tissue donors A pie chart is presented showing both the frequency (n) and the percentage of each cause of death among the corneal donors

The leading causes of death among corneal donors were cardiogenic shock (34; 18.4%), hypovolemic shock (31; 16.8%), acute respiratory failure (30; 16.2%), intracerebral hemorrhage (22; 11.9%), acute myocardial infarction (13; 7%), metabolic acidosis (10; 5.4%), and cerebrovascular accident (10; 5.4%) (Figure 4).

Donor deaths occurred in the following hospital areas: Internal Medicine (92; 49.7%), Emergency (67; 36.2%), Intensive Care Unit (20; 10.8%), General Surgery (5; 2.7%), and Orthopedics and Traumatology (1; 0.5%).

Seven (3.8%) from the 185 donors were excluded due to positive HIV tests. The median age of this group was 64 years (IQR 58−69). Among them, one (14.29%) was a female, and six (85.71%) were males. All excluded patients had comorbidities, including systemic hypertension (2; 28.57%), chronic kidney disease (2; 28.57%), chronic liver failure (2; 28.57%), and heart failure (1; 14.29%). No donors were excluded due to positive hepatitis B tests. Two (1.1%) from the 185 donors were excluded due to positive hepatitis C tests. No donors were excluded due to positive VDRL tests.

Seven (3.8%) from the 185 donors were excluded due to positive SARS-CoV-2 PCR tests. The median age of this group was 62 years (IQR 61−68). Of these, five (71.43%) were men and two (28.57%) were women. The comorbidities included type 2 diabetes mellitus (1; 14.29%), systemic hypertension (1; 14.29%), chronic kidney disease (2; 28.57%), heart failure (1; 14.29%), and chronic liver failure (2; 28.57%).

## Discussion

In our study, we observed a broad age range among cadaveric donors, spanning from two to 85 years, with a mean age of 56.34 years. The most prevalent age group was between 61 and 70 years, accounting for 31.35% of the donors. This finding contrasts with the epidemiological study by Merino-Cabrera et al., which reported a more restricted age range with the youngest donor being 15 years old [11]. Additionally, our results diverge from the data published by the Cuban Institute of Ophthalmology "Ramón Pando Ferrer" in 2014, where the most common age group was between 51 and 70 years, encompassing 51.18% of donors [12].

Comparing these findings with international studies reveals notable trends. For instance, the Corneal Retrieval Program in Kolkata, India, from 2011 to 2016, involved 4,300 donors with a mean age of 68.9 ± 13.4 years and an age range of 7.5 to 103 years. The predominant age group for donors was 60 to 80 years (55.8%) [13]. This is similar to our study's predominant age range, although it extends slightly older. The gender distribution in this study showed 57.4% male donors and 42.6% female donors, which aligns closely with our study's findings of 58.9% males and 42% females.

In contrast, the Eye Bank of Eastern India, which analyzed 743 corneal tissues from 373 donors between 2007 and 2011, reported a mean age of 52 ± 21 years with an age range of three to 95 years. The most common age groups were 41 to 50 and 71 to 80 years [14]. Although this study showed a younger average age compared to ours, the 71-80-year range overlaps with our predominant age group, albeit less frequently.

The Northern India Eye Bank, evaluating data from November 1999 to October 2015, assessed 1,646 corneas from 851 donors, with a mean age of 63.2 ± 19.5 years and an age range from 49 days to 102 years. The gender distribution was 58.3% male and 41.7% female, which closely matches our study [15]. However, the broader age range in this study contrasts with the more specific age ranges observed in our study.

In South America, the Brazilian National Institute of Traumatology and Orthopedics Eye Bank conducted a retrospective study of 839 donors from 2013 to 2021. This study found that 36.5% of donors were over 60 years old, with a male predominance of 58.2% [16]. This reflects a similar trend to our study, though the percentage of those over 60 is somewhat lower compared to our findings.

In New Zealand, the National Eye Bank study from 2000 to 2009 included 1,268 donors with a mean age of 67 years and an age range of five to 90 years. The gender distribution was 64% males and 36% females, and the median death-to-preservation interval was 18.5 hours [17]. While the mean age in New Zealand is older than in our study, the gender distribution is comparable.

Lastly, the University Medical Center Hamburg-Eppendorf in Germany conducted an extensive study from 1981 to 2010, involving 5,503 donors. The median age at death was 62 years, with a range of 49 to 72.5 years, and 64.8% of donors were males [15,18]. This study’s median age and gender distribution are consistent with our findings, although the mean age in our study is slightly lower.

A total of 16 corneal tissue donors were excluded due to positive results in the viral serological panel (HIV, VDRL, HBV, HCV, SARS-CoV-2), representing 8.6% of total donations. This seropositivity rate is notably higher compared to the findings of Patel et al., who reported a seropositivity rate of 2.32% among 947 cadaveric corneal donors, which includes infections such as HIV, hepatitis C, cytomegalovirus, and other microorganisms [19]. Our study's nearly fourfold higher rate suggests a significant prevalence of infectious diseases among potential donors at our institution, indicating that enhanced preventive measures in the general population might help reduce these rates and increase the pool of eligible donors.

Other ophthalmological centers report even lower seropositivity rates. For instance, the Eye Bank Association of Australia reports a rate of 0.92%, while RP Center, All India Institute Of Medical Sciences (AIIMS), New Delhi, reports 1.51%, M&J Institute two reports 1.19%, Eye Banks of America reports 4%, and Eye Bank, Canada, reports 1.21% [19]. These lower rates observed in various centers underscore a regional variability in seropositivity, potentially reflecting differences in population health and screening practices.

Regarding COVID-19, our study excluded corneal tissue from seven donors due to the detection of SARS-CoV-2 in nasal swabs, representing 3.8% of the total donors. Tello-Medina et al. noted that there is no confirmed evidence of SARS-CoV-2 transmission through ocular tissue and that positive PCR results from nasal swabs do not necessarily indicate the presence of the virus in corneal tissue [20]. Despite this, our institution's protocol mandates the exclusion of corneal tissue with positive PCR tests for SARS-CoV-2 to minimize the risk of transmission and ensure the safety of transplantation procedures.

In comparison, a study from the Regional Institute of Ophthalmology in Gujarat, India, reported that 2.2% of donors tested positive for infections, including HIV, hepatitis B, hepatitis C, and syphilis [13]. This rate is slightly lower than our study's rate but still highlights the significant issue of seropositivity in corneal donors. Another study found that 4% of donors had positive serologies for HIV, hepatitis B, or hepatitis C [17]. This data aligns closely with our observation that a substantial proportion of donors were excluded due to infectious diseases.

In our study, the leading causes of death among corneal donors were cardiogenic shock (34; 18.4%), hypovolemic shock (31; 16.8%), and acute respiratory failure (30; 16.2%). These findings reveal a significant focus on cardiovascular and respiratory issues. This contrasts sharply with the results reported by Merino-Cabrera et al., who identified cerebrovascular events as the primary cause of death, accounting for 10 (31%) cases. In our study, cerebrovascular events represented only 10 (5.4%) deaths [11].

Conversely, our findings align with those of Escalona et al., whose study (n = 590) also highlighted cardiac diseases as the leading cause of death (171; 29%), followed by cerebrovascular diseases (145; 24.7%) and respiratory diseases (90; 15.4%). The overlap in the top causes of death in our study with those reported by Escalona et al. suggests a common trend in the prevalence of cardiovascular and respiratory conditions among corneal donors [12].

A review from the Prasad Eye Institute in Hyderabad, India, covering 100 ocular tissues from September 2020 to April 2021, identified trauma (51; 51%) and suicide (33; 33%) as leading causes of death, with cardiogenic shock being less common [21]. This indicates a substantial regional difference, as trauma and suicide were more frequent causes compared to the primary causes in our study.

Further, another study by Ranjan et al. reported that the most common causes of death were cardiorespiratory failure (126; 34%) and traffic accidents (111; 30%). This study shows a significant emphasis on cardiorespiratory failure, aligning with our finding of acute respiratory failure being a prominent cause [14]. However, the high incidence of traffic accidents in their study contrasts with the types of death most frequently observed in our cohort.

In another analysis by Linke et al., 1530 (27.8%) patients died of natural causes due to old age, while 726 (13.2%) died from nonnatural causes, including accidents and poisoning. The remaining 3247 (59%) died from diseases, with cardiovascular diseases, sepsis, and liver cirrhosis being the top causes [15]. The prevalence of cardiovascular diseases in this study resonates with our findings but includes a broader range of conditions.

Lastly, a study examining the quality of procured tissue noted that the primary causes of death were cardiorespiratory failure, polytrauma, or suicides, with a noted decrease in endothelial cell density with increasing donor age [15,18]. Although this study did not mention the seropositivity of donors, the focus on cardiorespiratory and traumatic causes aligns with our study's emphasis on cardiovascular and respiratory issues, reflecting similar underlying health challenges among donors.

Regarding comorbidities, it is noteworthy that (45; 24.3%) of our patients had no comorbidities, a figure that contrasts with similar epidemiological profile studies, which report systemic hypertension rates in 48% of their donors [11]. However, the top three comorbidities were chronic kidney disease at 36 (19.5%), type 2 diabetes mellitus at 28 (15.1%), and systemic arterial hypertension at 31 (16.8%). This aligns with epidemiological reports, which show an increase in these conditions from 2010 to 2019, as these conditions are significant contributors to high morbidity and mortality and are major risk factors for preventable deaths [22].

While our study provides valuable insights into the characteristics and challenges associated with corneal tissue donation, limitations should be acknowledged. First, the use of a convenience sampling method, rather than a randomized approach, may introduce selection bias and limit the generalizability of the findings. The study's focus on a single hospital also restricts the applicability of the results to other settings or regions with different demographics and healthcare systems. The high seropositivity rate for viral infections observed in our study, while significant, may also reflect regional health issues or variations in screening practices rather than a universal trend. Addressing these limitations in future research through more comprehensive, multicenter studies and improved data collection methods could provide a clearer picture and help enhance organ donation practices.

## Conclusions

In conclusion, our study provides important insights into the characteristics of corneal tissue donors at General Regional Hospital 1 in Querétaro. The typical donor profile was predominantly male, with an average age of 56.34 years, who died from cardiogenic shock. This profile generally matches international data, showing a predominance of older age groups and male donors. However, our study found a notably higher seropositivity rate of 8.6% for viral infections, including HIV, hepatitis B, hepatitis C, and COVID-19, which is significantly higher than those reported in other countries. This highlights the need for improved health interventions and screening processes to enhance the safety and availability of corneal tissues for transplantation. Additionally, while broader causes of death, such as trauma and suicides, are noted in the literature, our study focused mainly on cardiovascular and respiratory causes, reflecting regional health issues. Effective coordination in organ donation and procurement within both public and private institutions is crucial for increasing national donation rates, as our country lags behind in meeting organ and tissue demands. Improving donation practices and raising public awareness are essential steps to address these challenges and enhance transplantation outcomes.
